# Relation between preoperative benzodiazepines and opioids on outcomes after total joint arthroplasty

**DOI:** 10.1038/s41598-021-90083-z

**Published:** 2021-05-18

**Authors:** Lisa V. Doan, Kristoffer Padjen, Deborah Ok, Adam Gover, Jawad Rashid, Bijan Osmani, Shirley Avraham, Jing Wang, Samir Kendale

**Affiliations:** 1 Department of Anesthesiology, Perioperative Care and Pain Medicine, New York University Grossman School of Medicine, 550 1st Ave., New York, NY 10016 USA; 2 Department of Neuroscience and Physiology, New York University Grossman School of Medicine, New York, NY USA

**Keywords:** Outcomes research, Therapeutics

## Abstract

To examine the association of preoperative opioids and/or benzodiazepines on postoperative outcomes in total knee and hip arthroplasty, we retrospectively compared postoperative outcomes in those prescribed preoperative opioids and/or benzodiazepines versus those who were not who underwent elective total knee and hip arthroplasty at a single urban academic institution. Multivariable logistic regression was performed for readmission rate, respiratory failure, infection, and adverse cardiac events. Multivariable zero-truncated negative binomial regression was used for length of stay. After exclusions, there were 4307 adult patients in the study population, 2009 of whom underwent total knee arthroplasty and 2298 of whom underwent total hip arthroplasty. After adjusting for potential confounders, preoperative benzodiazepine use was associated with increased odds of readmission (p < 0.01). Preoperative benzodiazepines were not associated with increased odds of respiratory failure nor increased length of stay. Preoperative opioids were not associated with increased odds of the examined outcomes. There were insufficient numbers of infection and cardiac events for analysis. In this study population, preoperative benzodiazepines were associated with increased odds of readmission. Preoperative opioids were not associated with increased odds of the examined outcomes. Studies are needed to further examine risks associated with preoperative benzodiazepine use.

## Introduction

Opioid prescribing rose steadily in the United States after 1999, peaking in 2010 and decreasing in 2011^1^. Opioid overdose deaths continued to increase in the United States from 1999 to 2015^2^. As underscored by the opioid epidemic, opioids may be associated with significant morbidity and mortality, even when used as prescribed^3–5^. Despite an increased awareness of risks and declining prescribing rates, in 2015 opioids were still prescribed at a rate approximately three times as high as in 1999^6^. In patients presenting for surgery, the prevalence of preoperative opioid use ranges from 8.8 to 35%, with higher rates in orthopedic populations^7–12^. Studies have shown preoperative opioid use or dependence was associated with poorer outcomes including increased length of stay, greater readmission rate, increased costs, and increased complications^7,8,10,11,13–16^.

There have also been rising trends in benzodiazepine prescriptions and overdoses in the United States, a danger overshadowed by the opioid crisis. The number of adults in the United States prescribed a benzodiazepine increased 67% from 1996 to 2013^17^. Opioids used in combination with benzodiazepines are particularly dangerous due to risks for sedation and respiratory depression. The number of patients prescribed both opioids and benzodiazepines is increasing, and in opioid overdose deaths, approximately 30% also involve benzodiazepines^18–20^. In 2016, the US Food and Drug Administration issued a boxed warning about the risks of concomitant opioid and benzodiazepine use. Preoperative benzodiazepine use is a risk factor for postoperative delirium, prolonged opioid use, and post-discharge drug related adverse events such as overdose or traumatic injury^21–23^. In a study from Iceland, preoperative prescriptions for both opioids and benzodiazepines were associated with increased 30-day and long term mortality in a mixed, noncardiac surgical cohort^24^.

Total knee arthroplasty (TKA) and total hip arthroplasty (THA) are the first and fifth most commonly performed surgeries in the United States, respectively^25^. Studies in total joint arthroplasty showed that those on preoperative opioids had higher readmission rates and poorer functional outcomes than opioid-naïve patients^8,13,16^. We hypothesized that patients undergoing TKA or THA prescribed opioids and/or benzodiazepines preoperatively had worse postoperative outcomes compared to those not on these medications.

## Methods

This study was approved by the Institutional Review Board at the New York University School of Medicine. All procedures were done in accordance with guidelines under the Institutional Review Board at the New York University School of Medicine. Consent was waived for this retrospective study by the New York University School of Medicine Institutional Review Board. This study is a retrospective analysis of an administrative database of adult patients who underwent primary TKA or THR between January 2013 and December 2014, at a single urban, academic institution. Data were obtained from our institutional electronic medical record (EMR) (Epic, Verona, Wisconsin) and included age, gender, surgical service, American Society of Anesthesiologists (ASA) physical classification score, race, body mass index (BMI), and emergency status, as described previously^26^. Presence of medical comorbidities (including congestive heart failure, atrial fibrillation, chronic obstructive pulmonary disease (COPD), dementia, diabetes mellitus, human immunodeficiency virus (HIV), hypertension, liver disease, coronary artery disease, chronic kidney disease, cancer, alcohol abuse, peripheral vascular disease, and hypothyroidism) was determined by presence of diagnosis in the EMR by International Classification of Diseases, Ninth Revision, Clinical Modification (ICD-9-CM) code at the time of surgery. Preoperative medication was determined from medication lists at admission.

Adult patients who underwent elective, primary TKA or THA requiring an inpatient stay between 2013 and 2014 were considered for inclusion in the study. Patients prescribed opioids and/or benzodiazepines preoperatively were compared to patients taking no opioids and/or benzodiazepines preoperatively. Preoperative opioid prescription and dose was verified by review of the pre-anesthesia evaluation note, which was completed by an anesthesiologist on the day of surgery and includes a home medication list. Postoperative opioid use was determined by manual review of charts. Oral morphine equivalent doses (MED) were calculated using morphine conversion factors per milligram of each opioid per day^27^. Patients on buprenorphine, naloxone, and/or naltrexone were excluded as these medications may uniquely alter postoperative opioid requirements. Patients admitted prior to the day of surgery were excluded as they may have received opioids acutely prior to surgery. Emergency surgeries were excluded as were those for acute fracture. Procedures with obviously erroneous data were not included. Patients who died in the hospital were excluded from analysis. During the study period, our institution’s postoperative pain management protocol for TKA and THA included oral acetaminophen around the clock and oxycodone as needed.

### Outcomes

The outcomes of interest included hospital readmission, respiratory failure, adverse cardiac events, infection, and hospital length of stay. Readmission was defined as any return to the hospital after discharge within 30 days. Respiratory failure was defined by presence of the corresponding ICD-9-CM code for respiratory failure or respiratory insufficiency. Adverse cardiac event was defined as presence of the ICD-9-CM codes for congestive heart failure, myocardial infarction, or atrial fibrillation^28^. Infection was defined as presence of ICD-9-CM codes for pneumonia, sepsis, or surgical site infection. ICD-9-CM codes used are provided in Supplemental Table S1. Adverse events were identified by new presence of codes during the admission. For hospital length of stay, day of surgery was considered day zero, and each day thereafter as one postoperative day.

### Statistical analysis

All descriptive values are presented as mean with standard deviation, median with interquartile range, or frequency and percentage as deemed appropriate. Normality was assessed using qq-plots and histograms. Comparisons between normal continuous data were performed using t-test or one-way ANOVA, between non-normal continuous data using Mann–Whitney or Kruskal–Wallis test, and those between categorical data using Chi-square or Fisher’s exact test. Missing data that were considered missing at random (MAR) were treated with multiple imputation, with number of iterations dictated by proportion of missing data, using the aregImpute function in the Hmisc package in the R statistical software (R Foundation for Statistical Computing, version 3.1.1, Vienna, Austria)^29,30^. Number of missing data points were as follows: BMI 226, type of anesthetic 416, preoperative MED 136.

Multivariable logistic regression was performed for the readmission, adverse cardiac events, infection, and respiratory failure outcomes, which were risk-adjusted for age, gender, ASA score, BMI, medical comorbidities, type of surgery, type of anesthetic, and postoperative MED as independent covariates. Comorbidities for inclusion were selected based on presence within the Charlson and Elixhauser comorbidity indices, which were defined using previously validated coding algorithms for ICD-9-CM comorbidity codes^31,32^. We assessed model covariates for collinearity and assessed for removal variables with variance inflation factors greater than four. All continuous data were assessed to be linear, and none were treated with splines in the final model.

Each outcome was analyzed with the interaction between long acting opioids, short acting opioids, and benzodiazepines as the exposure. Long acting opioids are inherently long acting or use a controlled release formulation. Long acting opioids included fentanyl patch, hydromorphone extended release (ER) methadone, morphine ER or controlled release (CR), oxycodone CR, oxymorphone ER, tapentadol ER, and tramadol ER. Short acting opioids included immediate release formulations of codeine, hydrocodone, hydromorphone, morphine, oxycodone, oxymorphone, tapentadol, and tramadol, alone or in acetaminophen or non-steroidal anti-inflammatory drug combinations. Odds ratios were calculated by coefficient exponentiation for the exposure variable of interest, along with 99% confidence intervals.

Multivariable zero-truncated negative binomial regression analysis that considered the count nature of the length of stay outcome and do not include the possibility of zero days length of stay was done, with the interaction between long acting and short acting opioids as the exposure, and which was risk-adjusted for age, gender, ASA score, BMI, type of surgery, type of anesthetic, expected length of stay and the previously defined medical comorbidities as independent covariates. Expected length of stay was determined by proprietary procedure-specific University HealthSystem Consortium (UHC) models utilized at our institution^33^. The UHC risk-adjusted calculation for expected length of stay is created from the data contributed by participating academic institutions. The relative weight assigned to each variable in the UHC length of stay calculation reflects the aggregate data from all hospitals. The UHC risk adjusted model for length of stay is proprietary; each of the 300 + models contains approximately 70–80 variables, is specific to a certain MS-DRG (Medicare Severity–Disease Related Group), and changes from year to year. Incidence rate ratios (IRR) were calculated by coefficient exponentiation for the exposure variable of interest, along with 99% confidence intervals. IRR values greater than 1 indicate an increase in risk of incurring additional time in the hospital. Multiple comparisons were adjusted for using Bonferroni correction with an adjusted p-value of less than 0.017 considered significant.

### Sensitivity analysis

As a sensitivity analysis, each outcome was analyzed with the interaction between preoperative MED and benzodiazepines as the exposure. E-values were calculated for statistically significant results to assess the magnitude of unmeasured confounders needed to explain away outcome associations^34^. A p-value of less than 0.05 was considered significant. As an additional sensitivity analysis, the length of stay model was constructed without the proprietary expected length of stay metric.

## Results

**Figure 1:**
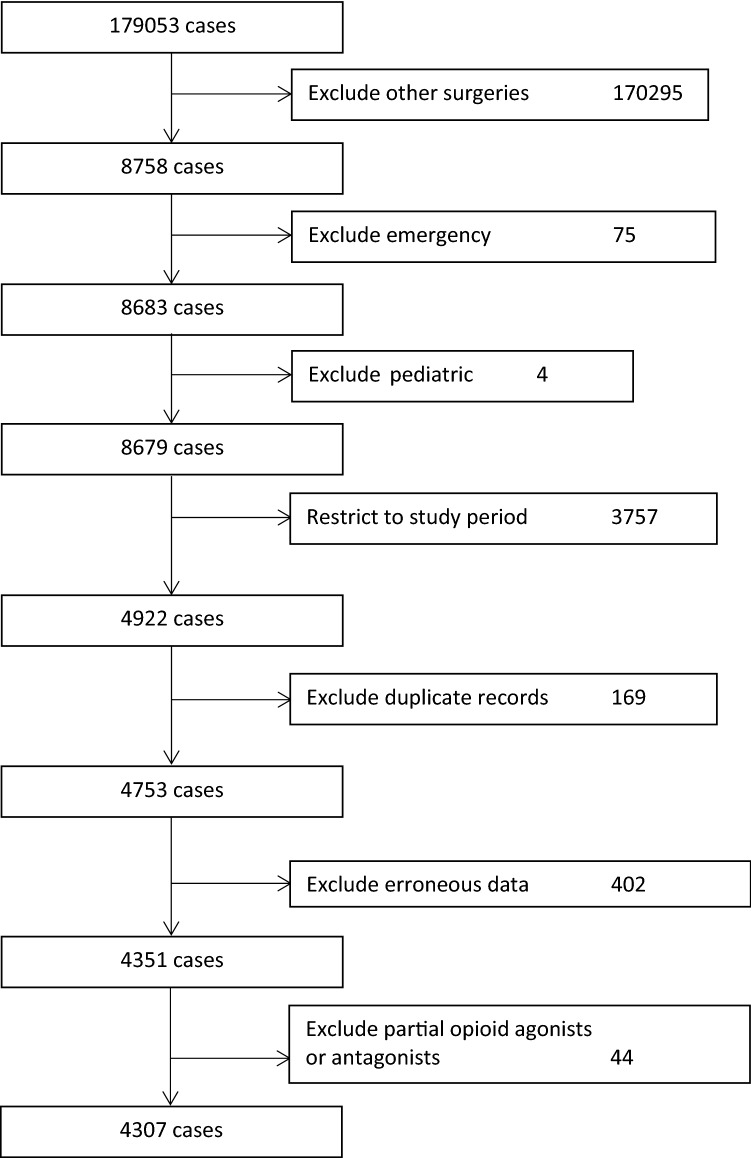
Flow diagram of inclusions and exclusions.

The entire dataset for the full date range included 179,053 cases. After limiting to elective TKA and THA in adults in the study period, there were 4922 cases. After excluding duplicate records, there were 4753 cases. After excluding 44 cases for patients on partial opioid agonists or antagonists and 402 cases with erroneous data, there were 4307 adult patients in the study population, of whom 2009 (47%) underwent TKA and 2298 (53%) underwent THA (Fig. 1). Patient characteristics are given in Table 1.

**Table 1:** Patient characteristics by preoperative medication.

	All patients (n = 4307)	On long acting opioid (n = 163)	On short acting opioid (n = 1007)	On benzodiazepine (n = 352)	On opioid + benzodiazepine (n = 154)
Age	64 (57–71)	61 (54–66)	63 (56–70)	64 (58–70)	62 (57–68)
Sex (Male)	1737 (40)	80 (49)	399 (40)	105 (30)	55 (36)
BMI	30 (26–34)	30 (26–35)	31 (26–35)	28 (25–34)	29 (25–35)
**ASA**					
I	209 (4.9)	3 (1.8)	29 (2.9)	6 (1.7)	1 (0.64)
II	2974 (69)	84 (52)	630 (63)	232 (66)	92 (60)
III	1069 (25)	73 (45)	338 (34)	109 (31)	58 (38)
IV	21 (0.49)	3 (1.8)	4 (0.40)	1 (0.28)	1 (1.1)
Unknown	34 (0.78)	0 (0)	6 (0.60)	4 (1.1)	2 (1.3)
**Race**					
White	2875 (67)	105 (64)	657 (65)	288 (82)	129 (84)
Asian	146 (3.4)	0 (0)	18 (1.8)	5 (1.4)	1 (0.65)
Black	602 (14)	27 (16)	163 (16)	15 (4.3)	5 (3.2)
Native American	8 (0.19)	0 (0)	1 (0.10)	1 (0.28)	0 (0)
Other	649 (15)	30 (18)	160 (16)	39 (11)	17 (11)
Unknown	27 (0.63)	1 (0.61)	8 (0.0.80)	4 (1.1)	2 (1.3)
CHF	53 (1.2)	0 (0)	12 (1.2)	3 (0.85)	2 (1.3)
Afib	199 (4.6)	1 (0.61)	59 (5.9)	16 (4.5)	8 (5.3)
COPD	159 (3.7)	18 (11)	48 (4.8)	20 (5.7)	12 (7.9)
DM	613 (14)	28 (1617	152 (15)	44 (13)	17 (29)
HTN	2278 (53)	80 (49)	579 (57)	171 (49)	75 (49)
CAD	429 (10)	28 (9.2)	106 (11)	33 (9.4)	12 (7.9)
PVD	113 (2.6)	9 (5.5)	34 (3.4)	8 (2.3)	3 (2.0)
Asthma	527 (12)	27 (17)	149 (15)	54 (15)	24 (16)
Smoking	384 (8.9)	40 (25)	135 (13)	44 (13)	26 (17)
General anesthesia	1078 (25)	68 (42)	322 (32)	102 (29)	52 (34)
Readmission	318 (8.7)	16 (9.8)	81 (8.0)	44 (13)	20 (13)
Respiratory failure	198 (4.7)	10 (6.1)	40 (4.0)	26 (7.4)	10 (6.6)
Length of stay	3 (2–3)	3 (2–3)	3 (3–4)	3 (3–4)	3 (3–4)

Age, BMI, and length of stay are given as median with interquartile range. Other values are number and percentages.

*BMI* body mass index, *Afib* atrial fibrillation, *ASA* American Society of Anesthesiologists, *CAD* coronary artery disease, *CHF* congestive heart failure, *COPD* chronic obstructive pulmonary disease, *DM* diabetes, *HTN* hypertension, *CAD* coronary artery disease, *PVD* peripheral vascular disease.

Preoperative opioids (without benzodiazepine) were present in 1107 (27%) of the study population, with 163 (3.8%) prescribed long acting opioids (with or without short acting opioids). Preoperative benzodiazepines were present in 506 (11.7%) of the study population, with concomitant preoperative benzodiazepines and opioids in 154 (3.6%) of the study population. For those on long acting opioids with or without short acting opioids, the median MED was 165 mg (IQR 60–300). For those only on short acting opioids, the median MED was 30 mg (IQR 18–60).

There was an association between preoperative benzodiazepines and readmission (OR 1.94 [99% CI 1.02, 3.45], p = 0.005), adjusting for age, gender, ASA score, BMI, medical comorbidities, type of surgery, type of anesthetic, and postoperative MED, with a calculated e-value of 3.2 (Table 2). There was no association between preoperative long acting (OR 1.32 [99% CI 0.29–4.02], p = 0.57) or short acting opioids (OR 1.16 [99% CI 0.77–1.73], p = 0.32) and readmission. There was no association between preoperative benzodiazepines and respiratory failure (OR 1.77 [99% CI 0.81, 3.52], p = 0.04). There was no association between preoperative long acting (OR 1.22 [99% CI 0.16–4.71], p = 0.74) or short acting opioids (OR 0.78 [0.44–1.32], p = 0.26) and respiratory failure. There was no association between preoperative long acting opioids (IRR 1.04 [99% CI 0.86–1.25], p = 0.62) or short acting opioids (IRR 1.02 [0.95–1.09], p = 0.52) nor preoperative benzodiazepines (IRR 1.08 [99% CI 0.96, 1.21], p = 0.16) and length of stay (Supplemental Table S2). There were no significant interactions between preoperative opioids and benzodiazepines for the examined outcomes. There were an insufficient number of infection and cardiac events for multivariate analysis.

**Table 2:** Regression results for readmission.

Variable	OR	99% CI	p-value
Preoperative long-acting opioid	1.32	0.29, 4.02	0.6
Preoperative short-acting opioid	1.16	0.77, 1.73	0.3
Preoperative benzodiazepine	1.94	1.02, 3.45	0.005
General anesthesia	0.97	0.67, 1.39	0.8
In hospital opioid use	1	1.00, 1.00	0.021
Hip vs knee replacement surgery	0.47	0.33, 0.65	< 0.001
Body mass index	1	0.98, 1.02	> 0.9
Age	0.99	0.98, 1.01	0.3
**Gender**			
Female		Reference	
Male	1.06	0.76, 1.46	0.7
**ASA score**			
I		Reference	
II	0.98	0.47, 2.27	> 0.9
III	1.01	0.44, 2.55	> 0.9
IV	0.45	0.01, 4.47	0.5
**Race**			
Asian		Reference	
Black	1.65	0.64, 5.26	0.2
Native American	2.82	0.03, 33.6	0.4
Other Race	0.99	0.38, 3.21	> 0.9
Unknown	0.59	0.01, 5.61	0.6
White	1.53	0.64, 4.69	0.3
**Medical comorbidity**			
CHF	1.53	0.38, 4.57	0.4
Afib	0.93	0.41, 1.90	0.8
COPD	1.03	0.44, 2.13	> 0.9
DM	0.85	0.52, 1.34	0.4
HTN	0.91	0.65, 1.27	0.5
CAD	1.33	0.76, 2.24	0.2
PVD	1.75	0.75, 3.63	0.066
Asthma	0.91	0.55, 1.44	0.6
Smoking	0.99	0.55, 1.66	> 0.9
Interaction long and short acting opioid	0.49	0.07, 3.16	0.3
Interaction long acting opioid and benzodiazepine	1.17	0.10, 10.6	0.9
Interaction short acting opioid and benzodiazepine	0.9	0.31, 2.45	0.8
Interaction any opioid and benzodiazpine	0.77	0.03, 19.4	0.8

*Afib* atrial fibrillation, *ASA* American Society of Anesthesiologists, *CAD* coronary artery disease, *CHF* congestive heart failure, *CI* confidence interval, *COPD* chronic obstructive pulmonary disease, *DM* diabetes, *HTN* hypertension, *OR* odds ratio, *PVD* peripheral vascular disease.

In the sensitivity analysis with preoperative MED as an exposure variable, there was not an association between MED and readmission (OR 1.00 [99% CI 0.99, 1.01], p = 0.34); preoperative benzodiazepines were associated with increased odds of readmission in this model (OR 1.84 [99% CI 1.03, 3.22], p = 0.005) with an e-value of 3. There was not an association between MED and respiratory failure (OR 1.00 [99% CI 0.99, 1.00], p = 0.22); preoperative benzodiazepines were not associated with respiratory failure in this model as well (OR 1.61 [99% 0.85, 2.84]; p = 0.04). When preoperative MED was used as the exposure variable, there was no association between preoperative MED and length of stay (IRR 0.99 [99% CI 0.99, 1], p = 0.09). However, preoperative benzodiazepines were associated with prolonged length of stay (IRR 1.09 [99% CI 0.99, 1.19], p = 0.014) with an e-value of 1.4. In the sensitivity analysis of length of stay model constructed without the expected length of stay metric, there was no association between preoperative long acting opioids (IRR 1.10 [99% CI 0.90–1.40], p = 0.11) or short acting opioids (IRR 1.00 [0.99–1.10], p = 0.05) nor preoperative benzodiazepines (IRR 1.10 [99% CI 0.99, 1.20], p = 0.03) and length of stay.

## Discussion

In this study from a single, urban academic institution of adults undergoing TKA and THA, preoperative benzodiazepines were associated with increased odds of readmission, adjusting for confounders. Preoperative opioids were not associated with increased odds of readmission. Neither preoperative opioids nor preoperative benzodiazepines were associated with increased odds of respiratory failure or increased length of stay, and there were no significant interactions between preoperative opioids and benzodiazepines for the examined outcomes.

Studies of benzodiazepines in the perioperative period have mainly focused on delirium and prolonged opioid use^21,22,35^. A few studies have examined the impact of preoperative benzodiazepine use on other postsurgical outcomes. In a study of patients undergoing general and orthopedic surgeries, preoperative benzodiazepines were associated with increased odds of a composite outcome of overdose, drug-related event, or traumatic injury within 30 days after surgery^23^. Length of stay has been examined in other studies. In a study of adult, noncardiac surgeries, we found that preoperative benzodiazepines were associated with increased hospital length of stay^36^. In a study of adult general, vascular, urologic, and plastic surgery procedures, preoperative anxiolytics were associated with increased length of stay and greater odds for a composite outcome of morbidity after surgery, including death, return to operating room, infection, renal insufficiency or failure, deep venous thrombosis or pulmonary embolism, stroke, and cardiac event^37^. In a study of patients undergoing minimally invasive transforaminal lumber interbody fusion, preoperative benzodiazepine use, but not opioid use, was associated with increased length of stay^38^. Readmission was not examined in these studies. In our primary analysis, preoperative benzodiazepines were not associated with prolonged length of stay. However, in the sensitivity analysis, preoperative benzodiazepines were associated with prolonged length of stay, and this association should be examined in future studies.

Some studies have examined the effects of known diagnosis of anxiety on postoperative outcomes. In a study of TKA and THA, a preoperative diagnosis of anxiety or depression was associated with higher odds of a composite outcome of postoperative morbidity, driven mainly by device-related complications and anemia. Costs for TKA, but not THA, were higher in patients with anxiety or depression^39^. In a large, population-based study of TKA and THA using the National Inpatient Sample, an inpatient health care database in the United States, those with anxiety had higher odds for increased length of stay and greater hospitalization costs. However, this study found lower odds of in-hospital mortality and cerebrovascular and cardiac complications in those with diagnosis of anxiety compared to those without anxiety or depression^40^. Preoperative medications were not included as covariates in these studies.

Interestingly, in this study sample, preoperative opioid use was not associated with increased odds of the examined outcomes. This contrasts with several studies examining preoperative opioid use and outcomes after total joint arthroplasty. In a study of patients undergoing primary TKA and THA, use of preoperative opioids was associated with increased odds of a composite outcome of complications within 90 days of operation in a multivariate analysis^8^. Readmission rates were not different between preoperative opioid users and nonusers. Length of stay was longer for preoperative opioid users in an unadjusted analysis. In a study using large, private insurance and Medicare claims databases, prolonged preoperative opioid use (> 60 days) was associated with increased odds of 30-day readmission compared to 0 to 60 days of use in patients undergoing TKA and THA, adjusting for confounders^13^. In a large study using a different administrative claims database, preoperative opioid users had increased risk of longer length of stay, 30-day readmission, and 90-day surgical site infection in patients undergoing TKA, THA, and total shoulder arthroplasty, adjusting for confounders^41^. Results from our study may differ from these due to differences in perioperative management, methodology, and sample population and size. In this study we have controlled for in-hospital opioid use whereas these prior studies did not^8,13,41^.

Few perioperative studies have examined preoperative opioid and benzodiazepine co-prescription and outcomes. In a recent retrospective study of noncardiac surgical patients, patients with preoperative prescriptions for both opioids and benzodiazepines or benzodiazepines alone had greater length of stay compared to those only prescribed preoperative opioids or neither medication. In a propensity score-matched cohort, patients with preoperative opioids only and preoperative benzodiazepines only had no difference in short- and long-term mortality compared to controls. In the group receiving concomitant prescriptions for preoperative opioids and benzodiazepines, there was an increased hazard of short- and long-term mortality compared to controls^24^. In the abovementioned study of preoperative benzodiazepines in general and orthopedic surgeries, there was an interaction between concurrent preoperative benzodiazepine and opioid prescription, with increased odds of adverse postoperative outcomes compared to either medication class alone^23^. Further study on preoperative opioid and benzodiazepine co-prescription is warranted.

A major limitation of our study is that causality cannot be determined. It is unclear if the findings related to preoperative benzodiazepine use in our study could be due to medication effect, such as withdrawal or drug interactions in postoperative period, or to underlying psychiatric illness. Anxiety, as noted above, has been associated with longer length of stay and postoperative morbidity. Anxiety in other studies has been found to be associated with high acute postoperative pain level, which could in turn lead to increased postoperative healthcare utilization^42,43^. Other limitations of this study include the possibility of residual confounding. Due to limitations in data structure, we were not able to determine the impact of duration of preoperative opioid or benzodiazepine use on postoperative morbidity. Morbidity was identified by the presence of ICD-9 codes. Thus, there may have been underreporting of certain complications. This was a single center study from an urban, academic institution, possibly limiting generalizability to other populations.

## Conclusions

In this study, we have shown that preoperative benzodiazepines are associated with increased odds of readmission in adults undergoing TKA and THA in an urban, academic institution whereas preoperative opioids were not associated with worse outcomes. More studies are needed to elucidate the impact of preoperative benzodiazepines and opioids in other settings and surgical populations, particularly as increasing numbers of adults are co-prescribed these medications.

## Supplementary Information

Supplementary Information.
