# Supplementary material for: Relation between preoperative benzodiazepines and opioids on outcomes after total joint arthroplasty
Source: Sci Rep. 2021 May 18;11:10528. doi: 10.1038/s41598-021-90083-z (PMC8131602; doi:10.1038/s41598-021-90083-z)
Supplement: Supplementary file 1 — Supplementary Information. [file 41598_2021_90083_MOESM1_ESM.pdf]

SUPPLEMENTARY MATERIAL

Relation Between Preoperative Benzodiazepines and Opioids on Outcomes after Total Joint Arthroplasty

Lisa V Doan, MD, Kristoffer Padjen, MD, PhD, Deborah Ok, MS, Adam Gover, MD, Jawad Rashid, MD, Bijan Osmani, MD, Shirley Avraham, MD, Jing Wang, MD, PhD, Samir Kendale, MD

Table 1. ICD-9-CM diagnosis codes used to identify major perioperative complications

Event	ICD-9 CM diagnosis code
Myocardial infarction	410
Atrial fibrillation	427.31
Congestive heart failure	428
Respiratory complications	518.81, 518.82
Infection	998.51, 998.59, 996.66

Variable	OR	99% CI	p-value
Preoperative long-acting opioid	1.04	0.86, 1.25	0.62
Preoperative short-acting opioid	1.02	0.95, 1.09	0.52
Preoperative benzodiazepine	1.08	0.96, 1.21	0.16
General anesthesia	1.07	1.01, 1.13	0.003
In hospital opioid use	1.00	1.00, 1.00	<0.001
Hip vs knee replacement surgery	0.99	0.94, 1.05	0.80
Body mass index	1.00	1.00, 1.01	0.001
Age	1.00	0.99, 1.00	0.44
Gender			
Female		Reference	
Male	1.04	0.99, 1.10	0.04
ASA score			
I		Reference	
II	0.74	0.59, 0.94	0.001
III	0.80	0.63, 1.01	0.02
IV	0.85	0.60, 1.20	0.21
Race			
Asian		Reference	
Black	1.03	0.89, 1.19	0.60
Native American	1.17	0.66, 2.08	0.47
Other Race	0.97	0.84, 1.13	0.64
Unknown	0.92	0.66, 1.29	0.54
White	1.03	0.90, 1.18	0.60
Medical comorbidity			
CHF	1.26	1.07, 1.49	<0.001
Afib	1.11	1.00, 1.25	0.01
COPD	1.07	0.95, 1.21	0.15
DM	0.94	0.87, 1.01	0.03
HTN	0.99	0.93, 1.05	0.65
CAD	1.11	1.02, 1.22	<0.001
PVD	1.15	1.00, 1.32	0.01
Asthma	0.94	0.87, 1.02	0.04
Smoking	0.91	0.83, 1.00	0.01
Expected length of stay	1.50	1.44, 1.56	<0.001
Interaction long and short acting opioid	0.78	0.61, 1.00	0.01
Interaction long acting opioid and benzodiazepine	0.59	0.38, 0.90	0.001
Interaction short acting opioid and benzodiazepine	0.97	0.80, 1.19	0.72
Interaction any opioid and benzodiazpine	2.34	1.37, 4.00	<0.001

Table 2. Regression results for length of stay. Afib = atrial fibrillation; ASA = American Society of Anesthesiologists; CAD = coronary artery disease; CHF = congestive heart failure; CI

= confidence interval; COPD = chronic obstructive pulmonary disease; DM = diabetes; HTN = hypertension; OR = odds ratio; PVD = peripheral vascular disease.
